# National Technology Validation and Implementation Collaborative (NTVIC): Guidelines for establishing Forensic Investigative Genetic Genealogy (FIGG) programs

**DOI:** 10.1016/j.fsisyn.2023.100446

**Published:** 2023-11-10

**Authors:** Ray A. Wickenheiser, Jennifer Naugle, Brian Hoey, Rylene Nowlin, Swathi A. Kumar, Mark A. Kubinski, Claire Glynn, Raymond Valerio, Lance Allen, Stephanie Stoiloff, Jennifer Kochanski, Christi Guerrini, Anne Marie Schubert

**Affiliations:** New York State Police Crime Laboratory System, Albany, NY, USA; Wisconsin Department of Justice Division of Forensic Sciences, USA; Missouri Highway Patrol Crime Laboratory Division, USA; Idaho State Police Forensic Services, USA; Verogen, a QIAGEN company, USA; Office of the Attorney General, State of Idaho, USA; University of New Haven, Henry C Lee Institute of Forensic Science, USA; Assistant District Attorney, Queens District Attorney's Office, USA; Colorado Bureau of Investigation Forensic Services, USA; Miami-Dade Police Department, Forensic Services Division, USA; Arizona Department of Public Safety, USA; Baylor College of Medicine, USA; Retired Sacramento District Attorney, USA

## 1 Foreword

On February 15, 2023, the National Technology Validation and Implementation Collaborative (NTVIC) Forensic Investigative Genetic Genealogy (FIGG) Technical Validation Working Group (TVWG) Subcommittee for Public Entity Policy and Procedure published an article in the journal Forensic Science International: Synergy entitled “National Technology Validation and Implementation Collaborative (NTVIC) Policies and Procedures for Forensic Investigative Genetic Genealogy (FIGG)” [1]. This document provided guidelines and considerations for public and private crime laboratories and investigative agencies exploring the establishment of a forensic investigative genetic genealogy (FIGG) program [1]. Stakeholders provided welcomed and valuable feedback for the subcommittee's consideration. Since the original publication, additional legislation has been introduced, or passed, and has been incorporated into this second version guidance document. As with the original version, while each jurisdiction is responsible for its own program policy, sharing guidelines to optimize resources, promoting practices and processes that enable the implementation of new forensic technology, and elevating quality are fundamental goals of the NTVIC [1].

This document was prepared by current members of the Policy and Procedures Subcommittee of the FIGG-TVWG, as follows:

**Table 1:** 

Member	Organization
Ray Wickenheiser, DPS (Chair)	Director, New York State Police Crime Laboratory System^a^
Jennifer Naugle	Deputy Administrator, Wisconsin Department of Justice Division of Forensic Sciences^a^
Brian Hoey	Director, Missouri Highway Patrol Crime Laboratory Division^a^
Rylene Nowlin	Idaho State Police Forensic Services^a^
Swathi A. Kumar, Ph.D	Director, Verogen (A QIAGEN company)^a^
Mark A.Kubinski, J.D	Special Deputy Attorney General, State of Idaho
Claire Glynn, Ph.D	University of New Haven, Henry C Lee Institute of Forensic Science^a^
Raymond Valerio	Assistant District Attorney, Queens District Attorney's Office
Lance Allen	Colorado Bureau of Investigation Forensic Services
Stephanie Stoiloff	Chief, Miami-Dade Police Department, Forensic Services Division
Jennifer Kochanski	Arizona Department of Public Safety
Christi Guerrini	Baylor College of Medicine
Anne Marie Schubert	Retired Sacramento District Attorney

a Denotes an original committee member.

This document has been approved by the National Technology Validation and Implementation Collaborative Steering Committee.

## 2 Scope

Forensic Investigative Genetic Genealogy (FIGG) utilizes an evidentiary single nucleotide polymorphism (SNP) deoxyribonucleic acid (DNA) profile generated by next generation sequencing (NGS) and established genetic and genealogical research methodologies to generate investigative leads in unsolved investigations such as missing and unidentified persons investigations and violent crimes [1,[2], [3], [4]]. On February 15, 2023, the NTVIC published an article entitled “National Technology Validation and Implementation Collaborative (NTVIC) Policies and Procedures for Forensic Investigative Genetic Genealogy (FIGG)” to summarize existing published guidance, including the Scientific Working Group for DNA Analysis Methods (SWGDAM) IGG Overview Document [5], the United States Department of Justice (US DOJ) Interim Policy [6], and the Maryland legislation [7]. The article also solicited feedback from the forensic community on a draft of proposed best practices and considerations for developing forensic genetic genealogy (FGG) SNP DNA profiles and conducting investigative genetic genealogy (IGG) in public laboratories and investigative agencies. Since the publication of the aforementioned paper, the state of Utah has passed the “Sherry Black Bill”, and the state of Florida has passed a bill exempting genetic genealogy materials from public records disclosure requirements [8]. In addition, multiple members of the forensic science, law enforcement, legal, and genealogical communities have provided extensive feedback on the draft guidelines.

Individual jurisdictions are responsible for establishing their own FIGG policies and procedures, overseeing its implementation and developing frameworks for evaluating program performance. The purpose of this paper is to provide a publicly available updated version that considers feedback from stakeholders and other interested parties regarding (1) compatibility with existing law, and (2) guidance for public and private-sector laboratories, investigative agencies and legal professions that may be developing state or local policies and procedures related to the establishment of a FIGG program. The term FIGG Responsible Authority (FIGG RA) is used herein to refer to the body responsible for the use and oversight of FIGG in a particular jurisdiction [1].

The scope of this document includes guidance on:

• Terminology and definitions used during the FIGG process
• Organizational approaches for establishing a FIGG Program
• Considerations for development of FIGG policy and procedures
  o case qualification and triaging
  o utilization of genetic genealogy databases
  o obtaining third-party DNA samples
  o obtaining putative perpetrator DNA reference samples
  o uses of certain genetic information and restrictions
  o privacy/protection of information
  o retention, release and expungement of information
• FIGG Education & training
• Quality Assurance and Quality Control (QA/QC)
• FIGG Case Reporting
• FIGG Oversight

## 3 Terminology and definitions

3.1 “Combined DNA Index System” or “CODIS”: the program operated by the Federal Bureau of Investigation to support criminal justice DNA databases and the software used to run the databases [9].
3.2 “Criminal proceeding”: the adversary judicial process prosecuted by a public officer and initiated by a formal complaint, information, or indictment charging a person with an offense denominated as criminal by applicable law and punishable by death, imprisonment, or a jail sentence [9].
3.3 “Direct-to-consumer (DTC) genetic genealogy services”: genetic genealogy services including databases that are offered by private companies directly to members of the public and investigative agencies rather than through clinical health care providers, typically via customer access to online websites [7].
3.4 “DNA”: deoxyribonucleic acid.
3.5 “DNA specimen” or “specimen”: a biological sample collected from an individual or a crime scene or collected as part of an investigation [7].
3.6 “DNA Technical Leader”: is an individual responsible for the oversight of the quality system in an accredited forensic DNA laboratory.
3.7 “Single nucleotide polymorphisms” or “SNP”: DNA sequence variations that occur when a single nucleotide (A, T, G or C) in a genomic sequence varies [7]. SNPs include variations that may be used to distinguish people for purposes of biological relationship testing or direct comparison purposes [1].
3.8 “Short tandem repeat” or “STR”: a genetic location in which the polymorphism or genetic variation is based primarily on the differences in the repeat elements among individuals. STRs are the genetic markers that are used primarily in local, state, and national DNA database systems [1].
3.9 “Familial DNA Searching” or “FDS”: a technique that deliberately searches for close biological relatives of a forensic STR DNA profile donor within an investigative database [10]. FDS typically is capable of determining close family relationships including immediate family members, i.e., parent-child, siblings.
3.10 “Forensic Investigative Genetic Genealogy” or “FIGG”: a procedure that combines genetic testing with traditional genealogical research to generate leads in investigations of unsolved violent crimes and unidentified human remains [1].
3.11 “Forensic Genetic Genealogy” or “FGG”: the process of developing a DNA SNP profile to be specifically uploaded into a genealogical database.
3.12 “Informed consent” as it pertains to third parties: consent based on disclosure of the purpose of third-party DNA sample collection and procedures for storing, transferring, and destroying the sample and associated data with an opportunity to ask questions and request clarification.
3.13 “Investigative Genetic Genealogy” or “IGG”: the investigative component of FIGG, that includes the upload of a SNP profile into a genealogical database, the creation of a family tree, and the investigation of leads [1].
3.14 “FIGG Responsible Authority” or “FIGG RA”: the body responsible for the conducting and oversight of FIGG in a particular jurisdiction” [1]. The FIGG RA may be determined by the jurisdiction within which FIGG will be applied or as determined by lawful authority.
3.15 “Forensic sample”: biological material collected from a crime scene, person, item, or location connected to the criminal event, that is reasonably believed by investigators to have been deposited by a putative perpetrator. A forensic sample also includes the biological material from unidentified human remains (UHR) [7].
3.16 “Putative perpetrator”: one or more criminal actors reasonably believed by investigators to have committed the crime under investigation and to be the source of, or a contributor to, a forensic sample deposited during or incident to the commission of a crime [7].
3.17 “Reference sample”: biological material collected from a known source [7].
3.18 “Third-party”: a person who is not a suspect in the investigation, however who may provide a lead through kinship association to identify the unknown donor of a forensic sample [7].
3.19 “Third-party DNA testing”: a DNA analysis conducted on a collected DNA sample from an individual with their consent who is not a suspect in an investigation, in an attempt to further genealogical research to identify the putative perpetrator.
3.20 “Unidentified human remains” or “UHR”: human remains from a person whose identity has not been established.
3.21 “Whole genome sequencing” or “WGS”: DNA analysis that determines the order of nucleotides (A, T, G or C) in an individual's entire genome.

## 4 Organizational approaches for establishing a FIGG program

Each jurisdiction should designate a FIGG RA to develop a framework for organizing, overseeing, and conducting a FIGG program based on their needs and legal requirements. Some models for consideration include:

4.1 Utilizing existing established processes and associated policies and procedures, namely, the laboratory (public or private) that maintains responsibility for DNA typing and/or database searching. Once an association or lead is made; law enforcement supports further investigation.
4.2 Designating a coordinating group consisting of all or a subset of representatives from law enforcement, the prosecuting attorney's office, and an authorized submitting investigative agency to facilitate communication and operations of FIGG.
4.3 Choosing a hybrid approach of 2.1 and 2.2.

## 5 Considerations for FIGG policy and procedures

Each state, region, and/or agency may develop their policies and procedures for implementation, in alignment with the individual jurisdiction's laws. A policies and procedures document may include, but is not limited to, the following.

### 5.1 Qualifying cases

Each jurisdiction should develop its criteria for case qualification, including exigent circumstances that may be evaluated on a case-by-case basis. The US DOJ Interim Policy and the Maryland and Utah laws governing FIGG include some case qualification considerations [[5], [6], [7]].

A. A violent crime as defined in the relevant jurisdiction [5]; or
B. Other violent crimes and/or defined by law and/or jurisdiction; or
C. Identification of a missing or unidentified human remains [7]; or
D. Post-conviction testing for crimes as defined in 3.1.A, B, and C above.

### 5.2 Case status

Current guidance suggests that reasonable investigative leads must be pursued prior to pursuing FIGG [1,6,7]. Each jurisdiction should develop its criteria for case management. Exceptions to a policy may be considered on a case-by-case basis, with FIGG being applied directly and/or concurrently with other investigative techniques such as when:

A. Biological evidence is limited, e.g., severely degraded or low input samples
B. A CODIS search did not yield a hit or was not possible because of the sample type (e.g., rootless hair), or
C. There is a significant and exigent risk to public safety.

### 5.3 Forensic samples

Forensic samples should be sufficient to develop a viable SNP DNA profile [1].

A. If a forensic sample has the potential to be consumed in analysis, consideration should be given to:
  i. Applying the most appropriate analytical technique to maximize the chances of obtaining useable data.
  ii. Delaying analysis as technology improves to enable a profile to be generated. The sensitivity of DNA analysis is rapidly developing, particularly for forensic samples. Therefore, sample preservation is critical to ensure the optimum opportunity for profile generation.
  iii. Re-examining case items for additional biological samples. Original forensic analysis traditionally did not aim to harvest all biological materials from evidence items, but rather to sample materials that may provide a result. Therefore, other items may possess additional biological materials that can be successfully analyzed to generate a SNP DNA profile.
B. Whole sample consumption is considered a last resort in forensic investigations, so must be given due consideration, have appropriate documentation and authorization, and be in compliance with federal and state laws prior to proceeding with analysis [*see Arizona v. Youngblood*, 109 S.Ct. 333 (1988)]. A separate request for whole sample consumption for FIGG purposes should be reviewed and authorized by the investigative agency, after considering input from the forensic laboratory and the prosecuting agency. All approvals from stakeholders should be retained within the case documentation. Developed policies for whole sample consumption shall be consistent with applicable law. Forensic laboratories may develop policies on consumption with their local prosecuting attorney or equivalent and provide them to submitting agencies.

### 5.4 Familial DNA searching (FDS)

Different ways to generate a lead via a DNA database include direct searching in CODIS, FDS or indirect searching in CODIS, and FIGG using public genetic genealogy databases. FDS uses STR DNA profiles and CODIS to develop investigative leads through a deliberate search for biologically related individuals [11]. An estimated 20 % of individuals in an investigative databank may be siblings [10]. FDS can be conducted independently from FIGG and does not require a SNP profile. Therefore, FDS should be considered (in jurisdictions permitting such searches) for unsolved cases where an STR forensic profile has been generated prior to pursuing FIGG.

Jurisdictions should develop triaging criteria to determine which database option(s) may be pursued, based on the sample type, the quantity/quality of DNA and the resulting STR DNA profile, the case circumstances, and the jurisdictional authority to use FDS, all of which should be considered prior to, simultaneously to, or independently of FIGG.

## 6 Program personnel and partners

### 6.1 Roles and responsibilities

Roles and responsibilities for those involved in a FIGG program may, include all or a subset of the following, based on the needs of individual jurisdictions:
  A. A designated laboratory official (DLO) [6];
    i. The DLO may be assigned by forensic laboratories and investigative agencies that are implementing FIGG.
    ii. The DLO's duties may include but are not limited to; assessing case eligibility, DNA sample and profile assessment, available SNP technology options, triaging mechanisms seeking authorizations for whole sample consumption, coordination of sample outsourcing to vendor laboratories (if applicable) and overseeing the upload/deletion of SNP DNA profiles from the database(s) following completion of a case.
    iii. The DLO may function as a single point of contact acting as a liaison between law enforcement, forensic laboratories, private laboratories, genealogical researchers, justice system officials, and other FIGG stakeholders.
    iv. The DLO provides information and education to key stakeholders, ensures compliance with laboratory policy and quality standards, maintains documentation of case records and recommendations, and performs other duties similar to those a CODIS administrator position currently requires [12].
  B. An administrative representative from the vendor sequencing laboratory, if used (DNA expertise) [6];
  C. An administrative representative with genealogical research expertise [1];
  D. An investigative agency representative with access to investigative databases (metadata) and crime analysis [1];
  E. A representative from the requesting investigative agency who can commit to surveillance and collection of covert samples in accordance with applicable laws and policies [1];
  F. A representative from the prosecuting agency that can provide legal expertise [1]; and
  G. A program/project lead [1].

### 6.2 Vendor laboratory vetting considerations

Considerations for evaluating a vendor laboratory partner for the generation of the SNP DNA profile for FIGG may include type and status of accreditation, scientific and technical capabilities, and case circumstances. When feasible and considering case circumstances, FIGG should be conducted in a laboratory that operates under a quality assurance system and is ISO accredited [1].

### 6.3 FIGG memorandum of understanding (MOU)

Prior to conducting FIGG, an MOU may be established between law enforcement and prosecutorial agencies to acknowledge that any investigative leads that are provided will be followed up, charges laid, and actively prosecuted, if warranted [1]. The MOU may also include confidentiality, security, and data handling provisions.

### 6.4 Genetic genealogy databases

A. FIGG shall only be conducted using publicly available genetic genealogy database(s) (e.g., GEDmatch PRO and FamilyTreeDNA) that explicitly permits law enforcement participation and allows customers to choose their own privacy options (opt-in/out) of law enforcement comparisons for FIGG.
B. Law enforcement agencies and forensic science service providers and their agents, including genealogical researchers and contractors, shall abide by genealogy database providers' terms of service and privacy policies, and the consumers self-declared privacy settings.
C. Law enforcement agencies and forensic science service providers recognize the role of genetic genealogy database providers as the responsible party to protect the privacy and confidentiality of all individuals within those databases, which includes individuals opting into law enforcement comparisons for FIGG, as well as those who choose to opt out. Law enforcement agencies and forensic science service providers and their agents, including genealogical researchers and contractors, will respect the privacy selections of all users, including those who choose to opt out of law enforcement comparisons for violent crimes using FIGG.
D. Law enforcement agencies and forensic science service providers and their agents, including genealogical researchers, shall immediately notify the genetic genealogy database service provider of any error in code or process which may inadvertently expose individuals whose selected privacy options do not meet the terms of use for opted-in/opted-out kits when performing law enforcement comparisons for FIGG. The genetic genealogy database service providers shall immediately address such errors with corrective actions to address the root causes identified.

### 6.5 Qualifications

As noted above, the case under investigation is the responsibility of law enforcement and/or prosecuting agency. Private laboratories and private FIGG practitioners/providers are subcontractors to those agencies and the FIGG RA. Therefore:

A. Qualifications of vendor laboratories will be determined by jurisdictional law and/or the FIGG RA with consultation of a DNA Technical Leader [7]. Determination of suitable qualifications may include accreditation status and compliance with jurisdictional requirements for processing forensic evidence.
B. The laboratory conducting the SNP DNA profile generation and the genealogist participating in FIGG shall be approved by the FIGG RA or prosecuting agency [7].
C. Qualifications of FIGG practitioners shall be determined by the FIGG RA [7]. Determination of suitable qualifications may include, but are not limited to, extensive FIGG experience, court deportment, and/or other qualifications that are reviewed and deemed suitable by the FIGG RA. FIGG Practitioners may be employed internally within an investigating agency or may be contracted externally with a private FIGG provider.

### 6.6 Quality assurance

A. Vendor laboratories shall maintain documentation regarding their Quality Assurance Systems to be provided upon request [1].
B. Vendor laboratories shall compare SNP DNA profiles against a staff elimination database for contamination checks prior to the release of the SNP DNA profile to the agency [1].
C. All laboratories conducting SNP sequencing for FIGG must be accredited. Acceptable standards include ISO-17025 or those deemed appropriate by the laws of the jurisdiction [1].
D. FIGG practitioners should have successfully completed a competency test approved by the FIGG RA prior to conducting any FIGG casework.
E. A quality assurance index search for contamination purposes may be conducted, including forensic and reference samples [1].

### 6.7 Training

FIGG education should be provided, by those with appropriate expertise, as follows.

A. When a case is initiated to enable members of the FIGG program to better assist with the investigation, and
B. With the release of FIGG investigative leads to investigators, as the lead may be a family member or not necessarily be the perpetrator,
C. For prosecutors and justice system officials, and
D. After the case is completed to all FIGG team members and the FIGG RA as lessons are learned [1].
E. Defined and documented training should be provided to each FIGG team member commensurate with their roles and responsibilities [1].

## 7 Processes

### 7.1 Third-party DNA samples

A third-party is an individual that is known not to be the individual who committed the crime, but rather one who may be genetically related and whose DNA sample may assist in building out further branches of a family tree or may directly lead to a candidate's identity through close relatives. General users (i.e., members of the public) populate the genetic genealogy databases, which are used to identify biological relative associations to forensic samples and can range from direct to close to distant relatives. These biological relatives provide a focal point from which to build out family trees. Through the building of family trees using genealogical records, a particular person may be reached in the tree(s) who it is believed may be genetically related to the forensic sample and may be a good candidate for third-party DNA testing.

The collection of a third-party DNA sample from a discarded item from an individual is legal, however, where possible, it is strongly encouraged to obtain consent from the third-party. A State, regional, or agency's FIGG Programs policy should take into consideration the ramifications of covert sample collection from third-party/non-suspect individuals on public participation in and perception of FIGG. Maintaining the trust and goodwill of the general public who populate the genetic genealogy databases is of paramount importance to the successful use of FIGG, and the covert collection of samples from third-party/non-suspect individuals could undermine the trust which is depended upon by law enforcement for successful FIGG cases.

Hence, the following non-exhaustive list of considerations may be evaluated with respect to the collection of third-party DNA samples to further genealogical research:

A. Genealogical investigation has built family trees; however, it has reached an impasse where additional information is not forthcoming to continue reliably building the trees, and
B. The collection of DNA samples from individuals identified through genealogical research could potentially breach the impasse by including or eliminating substantial portions of the family tree, and
C. There are no other reasonable means to breach the impasse by conducting by genealogical research without further DNA analysis, or
D. The amount of genealogical research required to breach an impasse is so large, or so intrusive as to render third-party DNA analysis the most reasonable approach.

### 7.2 Third-party DNA sample collection

When the need for a third-party DNA sample has met a jurisdiction or agency's criteria, the collection of a third-party DNA sample may be considered when:

A. Collection of a reference DNA sample from the third-party may provide additional leads to reach a candidate identification as noted above [6], and
B. Written informed consent should be provided by third parties prior to obtaining a DNA specimen, unless:
  i. There is a concern that obtaining an informed consent sample from a third-party may jeopardize the integrity of the investigation, provide notification of a putative perpetrator that may endanger public safety, or cause the putative perpetrator to flee. If any of those scenarios exist, then covert sample collection may be pursued, if in compliance with jurisdictional, state, and federal laws and policies.
  ii. If possible, prosecutor approval should be sought prior to a covert collection.
C. If a third-party denies providing an informed consent sample, consult with the prosecutor regarding potential next steps.
D. Third parties should only be contacted by law enforcement conducting the collection rather than genealogists or forensic laboratory personnel.
E. If overt collection of a third-party DNA sample is pursued, documented informed consent should be collected from the third-party [6].
F. It is advised that a Non-Disclosure Agreement (NDA) should be obtained so that the third-party does not discuss or distribute information regarding their participation in the case until provided with written notice that they may do so. An NDA may prevent interference in an ongoing investigation.
G. It is also advised that the law enforcement officer/agent collects genealogical/family tree information from the third-party during interview to compare with the prior genealogical research conducted in the investigation.
H. When a third-party has consented to participation, the third-party should be provided with options for how they can provide their DNA sample/data, and the relevant privacy options explained, which may include:
  i. If the third-party has previously taken a Direct-To-Consumer (DTC) DNA test (e.g., AncestryDNA, etc.), a request can be made to the third-party to voluntarily upload their DNA data file to the genetic genealogy database(s) themselves, or they may provide their downloaded DNA data file to the investigating agency for one-to-one comparisons to the forensic unknown DNA data [1]. Instructions should be provided to the third-party on how to download their DNA data file, and how to upload to the relevant genetic genealogy database(s). The relevant privacy options should also be explained regarding opting in/out for law enforcement comparisons. Third-parties must never be requested to provide access/log-in details to their DTC DNA test results at consumer DNA sites (e.g., AncestryDNA, etc.)
  ii. If the third-party has not previously taken a DTC DNA test, they may be offered to be supplied with a DTC DNA test (e.g., FamilyTreeDNA) by the investigating agency. The third-party should be informed of all the possible benefits and/or risks taking a consumer DNA test can have (e.g., matching to previously unknown close biological relatives, etc.). DTC DNA test results typically require 2–8 weeks to be processed. Once the results are ready, the third-party should be provided with further instructions to download/upload their DNA data file.

If the third-party wishes to participate, however does not want to take a consumer DNA test, a buccal sample can be collected from the third-party for SNP sequencing analysis to generate a SNP profile for upload and comparison to the genetic genealogy database(s). The SNP profile should be uploaded as a “law enforcement upload” through the relevant portals/procedures to ensure their profile is not visible to any general users [1].

### 7.3 Putative perpetrator DNA samples

A. A putative perpetrator DNA sample that is collected covertly may be analyzed for a short tandem repeat (STR) DNA profile to determine if it matches an STR DNA profile obtained from the forensic sample.
B. If judicial proceedings will be initiated, a direct reference sample should be obtained from the suspect by any legal means including, for example, upon arrest, with a search warrant, or with a court order. This direct reference sample should use STR analysis to confirm it matches an STR DNA profile obtained from the forensic sample.
C. In the event that an STR DNA profile is not available for the forensic sample, a SNP profile may be generated for comparison between the reference sample and the forensic sample for the purposes of identification.
  i. In the event an STR DNA profile is not available, and a SNP profile is used for comparison to the forensic profile, the SNP data should not be used “to determine the sample donor's genetic predisposition for disease, any other medical conditions, psychological trait” [6,7], nor genealogical, medical, or scientific research of any kind.

### 7.4 Uses of genetic information

A. Since the search and seizure of biological material abandoned at a crime scene is lawful, a forensic sample may be subjected to a wide variety of genetic analyses which can be developed to identify the donor, including phenotypic information and genetic ancestry.
B. Forensic samples may be analyzed to provide potential eye color, hair color, skin color, and physical traits such as age estimation for the purpose of investigative intelligence.
C. There shall be no use of reference samples beyond indirect and direct comparison utilizing STRs or SNPs. As by their definition, reference samples come from known individuals, hence there is little to be gained by use of these samples beyond comparison of some genetic markers to the forensic sample for the purposes of identification only. At the same time, the potential for other uses to undermine public trust is high.
D. No data generated from the biological samples subjected to FIGG analysis, whether the forensic sample or third-party samples, may be used for other purposes such as “to determine the sample donor's genetic predisposition for disease, any other medical conditions, psychological trait” [6,7], nor genealogical, medical, or scientific research of any kind.

### 7.5 Data management and security

A. The case under investigation is the responsibility of law enforcement and/or the prosecuting agency and not the vendor laboratory, nor the FIGG practitioner/provider who is performing research or analysis on the case. Therefore, responsibility for all documentation and data rests with the investigative authority and the FIGG RA.
B. Data storage and transfer
  i. DNA data and case information should be stored in accordance with CJIS policies. A secure method for data storage, transfer, or exchange such as a secure file transfer protocol (SFTP) site should be established.
  ii. The FIGG RA should include policies that cover transitions, in the event a case is transferred from one laboratory or genealogical research entity to another.
  iii. Upon successful completion of the FIGG investigation as deemed by the FIGG RA (e.g. final judgment in a criminal case; or if no charges result, identification of a suspect or UHR confirmed by other means), “the [genealogist] participating in the [FIGG] shall turn over to the investigator all documentation, records, and materials collected, including material sourced from public records, family trees constructed, and any other genetic or nongenetic data analyses collected” [6,7].
  iv. If court testimony or additional analysis or research is required, appropriate documentation should be provided by the FIGG RA to permit analysis, investigation, preparation, and testimony as necessary.
C. Data retention and deletion
  i. The retention and deletion of all FIGG data/documentation/records/trees built during a FIGG investigation must adhere to the corresponding state and/or federal law.
  ii. The FIGG practitioner/provider or vendor laboratory “may not keep any records or materials in any form, including digital or hard copy records” [7] unless legally required, as required by the agency's retention policy, and/or by a criminal justice agency [6,7] or FIGG RA.
  iii. The genealogist or investigative agency shall ensure that all records have been deleted or removed from any website/platform where the FIGG analysis/research was developed e.g., family trees built in platforms such as Ancestry.com or Lucidchart [6,7]. Transfer of ownership/log-in credentials for such sites must be performed.
  iv. If the DNA analysis was enabled by a third-party taking a direct-to-consumer (DTC) DNA test (e.g., AncestryDNA, etc.), and the third-party voluntarily provided their DNA data file for upload to the genetic genealogy database(s), then only the DNA analysis data possessed by the FIGG RA, investigating authority, forensic laboratory and genealogist will be destroyed.
  v. A person, agency or laboratory may not willfully retain or fail to destroy genetic genealogy information, SNP DNA profiles, DNA samples or DNA data generated during the course of the FIGG process that are required to be destroyed [7].
  vi. Once the FIGG has been concluded as determined by the FIGG RA, deletion or retention of third-party DNA samples and all associated data from their DNA analysis may be determined based on jurisdictional law or the agency's policy [6,7].
D. Data disclosure and confidentiality
  i. No person should disclose genetic genealogy data, SNP DNA profiles, or DNA samples except where authorized by law or order of a court of competent jurisdiction or policies [6,7].
  ii. Identifying information of all third parties should be kept strictly confidential [[6], [7], [13]].
  iii. The prosecuting agency “shall retain and disclose any records or material as required under the [applicable state and federal regulation, the rules of discovery, or other court orders,] but may not otherwise use or share the records or materials” [6,7].
  iv. Neither the laboratory conducting SNP or other DNA analysis, nor a law enforcement official or a genealogist may disclose genetic genealogy information or details associated with an ongoing investigation without authorization from the prosecuting agency [6,7].
  iv. Consideration should be given by genealogical researchers to build family trees outside of public databases and their associated tools, as privacy, security, and confidentiality is not assured. For example, family trees in ancestry.com set to private are not assured to be so, therefore use of programs that reside on the private computer of a genealogical researcher are preferable.
  v. Personally identifiable third-party information may not be included in warrants and other legal documents which could reveal the identity of related individuals [1] noting these individuals are not suspects in the instant case and their privacy should be protected.

### 7.6 Discovery

A. FIGG discovery must be provided in accordance with relevant law.
B. As innocent individuals are documented in family trees through genealogical research that links a SNP DNA profile to biological relatives, who in turn are linked to the suspect, privacy interests of these individuals should be protected in compliance with jurisdictional law.
C. If discovery is provided that includes identifying information associated with third-party individuals included in family trees, attempts may be made to protect this information such as with a court order.
D. In the event there is no forensic STR DNA profile for a direct match to a suspect reference sample, a SNP panel will be required for comparison between a suspect reference sample and the forensic sample(s). It is anticipated that this situation will more likely require presentation in court and discovery should be provided in accordance with relevant law. For cases that do not require presentation in court, reports shall include sufficient information to be deemed informative by all interested parties.
E. Adherence to policies and procedures can be enforced via procedures such as those provided by the FBI Quality Assurance Standards to ensure CODIS compliance [12].

### 7.7 Reporting

Data that can aid in the continuous improvement of the FIGG program should be collected [6,7]. Data may include the following.

A. Number of FIGG cases investigated [7];
B. Type of crime for which the FIGG case is being investigated;
C. DNA specimen details including type of specimen (biological material), DNA quantity and quality;
D. Type of DNA typing and analysis applied to the sample specimen;
E. Identity of vendor laboratories conducting SNP sequencing analysis and FIGG practitioners/providers conducting genealogical research;
F. Number of FIGG cases entered into genetic genealogy databases;
G. Number of leads resulting in an identification [6,7];
H. Costs of FIGG procedures (both cumulative and categorized by laboratory workflow (in-laboratory or vendor), genealogy, investigation [6,7];
I. “Number of times a third-party DNA sample was requested and collected” [7];
J. “Number of [FIGG] requests made by defendants and post-conviction attorneys” [7]; and/or
K. The case outcomes of each FIGG investigation [6,7].

### 7.8 Oversight

The FIGG RA, forensic laboratories, and investigative agencies implementing FIGG may choose to consult with a panel, a commission, or a committee. The panel, commission, or committee shall be comprised of stakeholders deemed appropriate by the relevant jurisdiction.“A panel comprised of judges, prosecutors, defense attorneys, public defenders, law enforcement officials, crime laboratory directors, bioethicists” [7], genealogists, “racial injustice experts, criminal justice researchers, civil and privacy rights organizations, and organizations representing the families impacted by the criminal justice system” [7], including victims’ rights advocates, “may be convened to review the annual report each year and make policy recommendations” [7].

## Declaration of competing interest

The authors wish to declare the following competing interests: S.A.K is a current employee and shareholder of QIAGEN Inc. C.J.G. and R.A.W. are members of the Investigative Genetic Genealogy Working Group of the Scientific Working Group on DNA Analysis Methods. C.J.G is a member of the Advisory Board of the Investigative Genetic Genealogy Accreditation Board.
